# Investigating the role of depression in obstructive sleep apnea and predicting risk factors for OSA in depressed patients: machine learning-assisted evidence from NHANES

**DOI:** 10.1186/s12888-025-07414-x

**Published:** 2025-10-10

**Authors:** Xiangyang Cheng, Fang Liu, Xiao Zhang, Ye Liu, Jiaxi Guo, Xuelai Zhong, Dongdong Tian, Aijie Pei, Xuwu Xiang, Yongxing Yao, Diansan Su

**Affiliations:** Department of Anesthesiology, The First Affiliated Hospital, Zhejiang University School of Medicine, 79 Qingchun Road, Hangzhou, 310003 China

**Keywords:** Depression, Obstructive Sleep Apnea, Nhanes, Machine learning

## Abstract

**Objective:**

The relationship between depression and obstructive sleep apnea (OSA) remains controversial. Therefore, this study aims to explore their association and utilize machine learning models to predict OSA among individuals with depression within the United States population.

**Methods:**

Cross-sectional data from the American National Health and Nutrition Examination Survey were analyzed. The sample included 14,492 participants. Weighted logistic regression analysis was performed to examine the association between OSA and depression.Additionally, interaction effect analyses were conducted to assess potential interactions between each subgroup and the depressed population.Multiple machine learning models were constructed within the depressed population to predict the risk of OSA among individuals with depression, employing the Shapley Additive Explanations(SHAP) interpretability method for analysis.

**Results:**

A total of 14,492 participants were collected. The full-adjusted model OR for Depression and OSA was (OR,1.31;95%CI(1.08, 1.60); *P* < 0.005).The positive association between depression and OSA was revealed in all models.The interaction analysis revealed no subgroups exhibited statistical significance. The Neural Network was identified as the best-performing model, achieving the highest Youden's Index, AUC, and Kappa scores. SHAP analysis highlighted the most significant predictors of OSA: BMI, Age, Marital status, Hypertension, Caffeine intake, Sex, Alcohol status, and Fat intake.

**Conclusion:**

In conclusion, our research indicates that depression is associated with OSA, highlighting the importance of early detection and management of depressive symptoms in individuals at risk of OSA.ML models were developed to predict OSA and were interpreted using SHAP. This method identified key factors associated with OSA, encompassing demographic, dietary, and health-related dimensions.

**Supplementary Information:**

The online version contains supplementary material available at 10.1186/s12888-025-07414-x.

## Introduction

Obstructive sleep apnoea is a common disorder of repetitive pharyngeal collapse during sleep [1].Due to the multifactorial nature and societal consequences of obstructive sleep apnea, the disease is associated with a high economic and social burden. In 2015, the cost of diagnosing and treating obstructive sleep apnea in the United States was approximately $12.4 billion [2].In a study on the global prevalence of obstructive sleep apnea, nearly 1 billion people are affected by obstructive sleep apnea (OSA), with prevalence rates exceeding 50% in some countries [3].Evidence suggests that OSA is a significant factor contributing to adverse health outcomes, and treating this condition is generally beneficial for minimizing associated negative clinical outcomes and improving sleep-related quality of life [4]. Meanwhile,Evidence indicates that OSA is independently associated with increased likelihood of hypertension,cardiovascular disease, stroke [5].

Depression is one of the most serious mental health disorders and a leading cause of mortality worldwide [6]. Globally, the number of reported cases of depression rose significantly from 172 million in 1990 to 258 million in 2017, marking a 49.86% increase [7]. Between 2015 and 2019, the prevalence of depression continued to rise across diverse populations [8].

The relationship between obstructive sleep apnea and depression, however, remains a subject of debate [9–16]. Current evidence is insufficient to establish a conclusive link between the two conditions. Meta-analyses have demonstrated comparable improvements in depression scores between CPAP and surgical interventions for OSA, providing robust evidence for the efficacy of surgical treatment on OSA-related mood disorders [17]. Furthermore, a bidirectional relationship between depression and OSA has been well documented [18].Moreover,study demonstrate that the potential mechanism is Inflammatory factors and oxidative stress [19–23]. However, certain meta-analyses have failed to establish this significant association [16]. To address this gap, our study aims to conduct a detailed cross-sectional analysis using data from the National Health and Nutrition Examination Survey (NHANES) for the periods 2005–2008 and 2015–2018. Specifically, we seek to investigate the association between depression—alongside various lifestyle and dietary factors—and OSA.While prior studies have examined OSA-depression associations using traditional statistics, no large-scale population-based research has employed machine learning (ML) to identify and interpret OSA predictors specifically within depressed individuals—a gap our study addresses.

## Methods

### Study data population

The National Health and Nutrition Examination Survey (NHANES) is a research program designed to evaluate the health and nutritional status of adults and children in the United States. All NHANES data are publicly available and can be freely accessed at:https://wwwn.cdc.gov/nchs/nhanes/Default.aspx. These data are widely used in epidemiological studies and health sciences research, contributing to the development of evidence-based public health policies, the design of health programs and services, and the advancement of health knowledge for the nation.

**Fig. 1:**
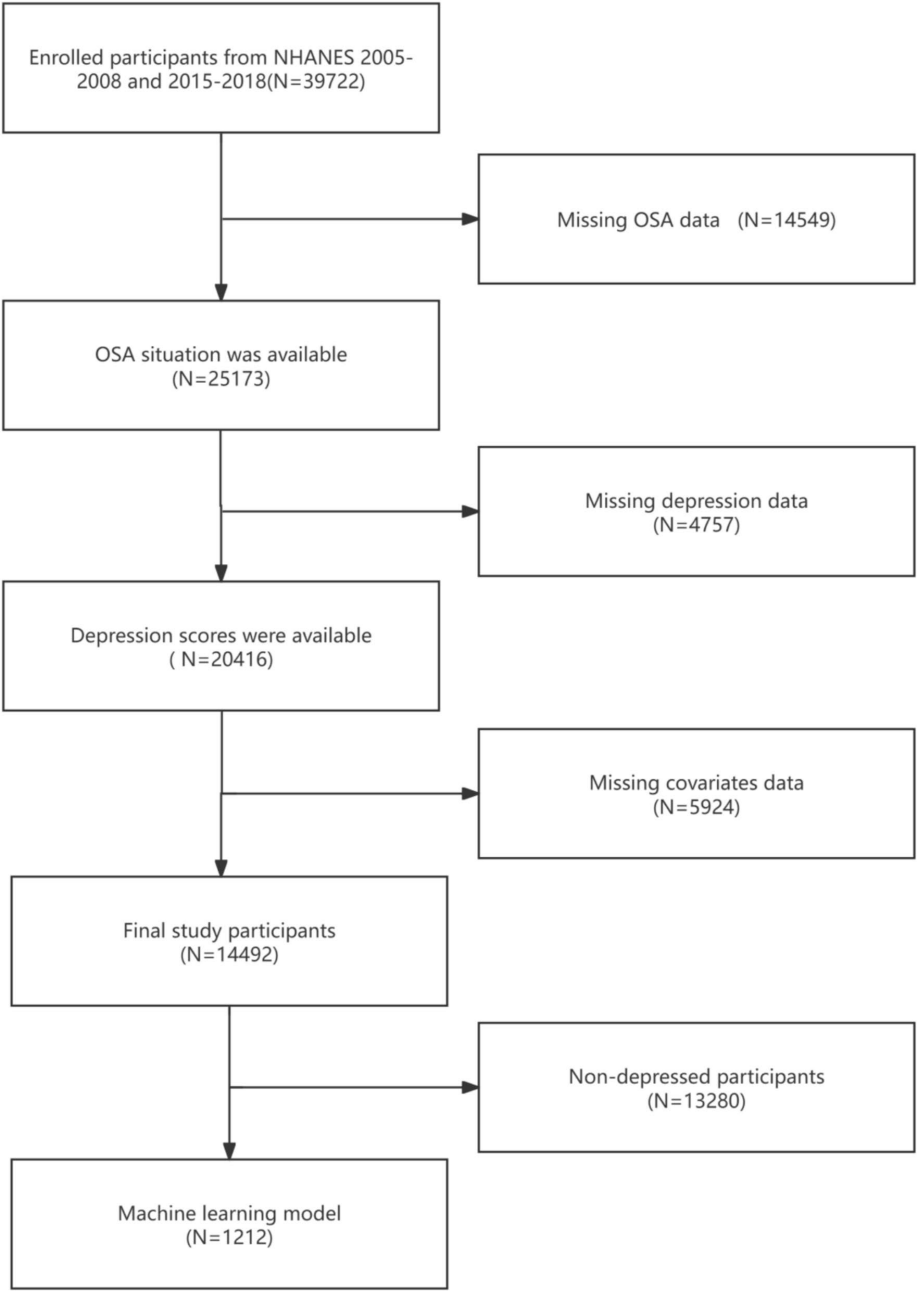
Flow chart of participants from NHANES included in the study

We included data from four NHANES cycles. The originally enrolled participants in each cycle were as follows: 2005–2006 (*N* = 10,348), 2007–2008 (*N* = 10,149), 2015–2016 (*N* = 9,971), and 2017–2018 (*N* = 9,254). The initial sample comprised 39,722 individuals. First, we excluded 14,549 participants with missing obstructive sleep apnea (OSA) assessment data, leaving 25,173 participants who completed OSA evaluations. Next, 4,757 participants without valid depression scores were excluded, resulting in 20,416 participants with depression data. Finally, we excluded 5,924 participants with incomplete covariate information. After these successive exclusions, 14,492 participants with complete data remained. These participants were distributed across four NHANES cycles as follows: 2005–2006 (*N* = 3,445), 2007–2008 (*N* = 3,904), 2015–2016 (*N* = 3,538), and 2017–2018 (*N* = 3,605).From the final sample (*N* = 14,492), depressed participants (*N* = 1,212) were selected for machine learning modeling, as detailed in Fig. 1.

### Independent variables: OSA

OSA status was determined using questionnaire data related to sleep habits and disorders, as described in a prior study [24]. Participants responded with either "yes" or "no" to a series of questions. Based on relevant research [25], OSA was defined by meeting any of the following criteria:Snoring three or more times per week;Experiencing wheezing, snoring, or breathing cessation at least three nights per week;Feeling excessively drowsy during the day on more than 16 to 30 occasions, despite obtaining seven or more hours of sleep nightly. Insomnia was defined based on the survey question: 'Ever told doctor had trouble sleeping?' (Yes/No).

### Dependent variable: depressive symptoms

The severity of depression was evaluated using the Patient Health Questionnaire-9 (PHQ-9) [26]. A score of ≥ 10 was used as the threshold, as it optimizes sensitivity and specificity across both overall and subgroup analyses. The PHQ-9 consists of nine questions designed to assess depressive symptoms. Responses are scored based on symptom frequency, with 0 indicating “not at all,” 1 for “several days,” 2 for “more than half of the days,” and 3 for “nearly every day.” Total scores range from 0 to 27, with a score of ≥ 10 serving as the clinical cutoff for depression diagnosis in primary care settings. In this study, PHQ-9 scores were a key variable of interest.

### Covariate variables

Demographic information collected included age, sex, race, marital status, education level, poverty-to-income ratio (PIR), lifestyle factors (alcohol status, smoking status), medical conditions (hypertension, diabetes), dietary intake (fat, fiber, and caffeine intake), and body mass index (BMI).

Race: Categorized as Non-Hispanic White, Non-Hispanic Black, Mexican American, Other Hispanic, and Other.Marital Status: Classified into six groups: divorced, living with a partner, married, never married, separated, and widowed.Education Level: Grouped into three categories: below high school, high school, and above high school.Poverty: Based on PIR, participants were classified into three groups: ≤ 1.0, 1.0–3.0, and > 3.0.Lifestyle Factors:Alcohol Use: Defined as a "yes" response to either of the following questions: “Have you ever had 5 or more drinks every day?” or “Have you ever had 4/5 or more drinks every day?”Smoking Status: Participants who answered "YES" to the question, “Have you smoked at least 100 cigarettes in your lifetime?” were classified as smokers.

Medical Conditions:Hypertension: Defined as a "YES" response to either of the following questions: “Have you ever been told you have high blood pressure?” or “Are you currently taking prescription medication for hypertension?”Diabetes: Participants were considered to have diabetes if they reported a professional diagnosis or had an HbA1c level exceeding 6.5% (47.5 mmol/mol).Dietary Intake: fiber, fat, and caffeine intake data were derived from the average of two-day dietary recall data.BMI:BMI was calculated as weight in kilograms divided by height in meters squared and categorized into four groups [27]: underweight (BMI ≤ 18.5), normal weight (18.5 < BMI ≤ 25), overweight (25 < BMI ≤ 30), and obese (BMI > 30).

### Statistical analysis

All statistical analyses accounted for the complex sampling design of the NHANES database, with individual sample weights determined by WTDRD1/4. Frequency and percentage analyses were stratified by OSA status.Continuous variables with non-normal distributions were described using median and interquartile range (IQR).Categorical variables were summarized as sample counts with their corresponding weighted percentages.The Kruskal–Wallis test was used to compare continuous variables, while the Rao-Scott chi-squared test was applied to assess weighted percentages of categorical variables across the entire population.Weighted logistic regression models were employed to examine the relationship between OSA and depression.Model 1: Unadjusted (crude model).Model 2: Adjusted for age, sex, race, marital status, education level, and PIR.Model 3: Further adjusted for alcohol use, smoking status, BMI, daily fat intake, daily fiber intake, daily caffeine intake, hypertension, and diabetes.All regression models incorporated survey weights. Additionally, interaction effect analyses were conducted to evaluate potential interactions between subgroups and the depressed population.And we use take some sensitivity analysis to ensure robust result.Multiple Imputation and Inverse Probability Weighting to deal with missing data.

### Development and validation of machine learning models

This study employed a Least Absolute Shrinkage and Selection Operator (LASSO) regression model to investigate the association between various research variables (excluding diagnostic variables) and the risk of OSA in individuals with depression. LASSO regression controls model complexity by introducing a regularization coefficient (λ) and compresses the coefficients of less relevant features to zero through L1 regularization, thereby achieving variable selection and dimensionality reduction. The model was optimized using ten-fold cross-validation, with λ.min selected as the optimal regularization parameter.

Eight variables were ultimately selected to construct a machine learning model tailored for the depressed population: BMI, age, marital status, hypertension, daily caffeine intake, sex, alcohol use, and daily fat intake.

To develop an OSA risk prediction system and evaluate its performance, the dataset was split into training and validation sets at a 7:3 ratio. Seven machine learning models were constructed, including Decision Tree (DT), Random Forest (RF), Extreme Gradient Boosting (XGBoost), K-Nearest Neighbors (KNN), Logistic Regression (LG), Support Vector Machine (SVM), and Neural Network (NNET), alongside a multifactor logistic regression model (LR) as a control. NNET model data were standardized using Z-score normalization.

For the validation set, the discriminative ability of the models was systematically evaluated using Receiver Operating Characteristic (ROC) curves and confusion matrices. Performance metrics such as the Area Under the Curve (AUC), Youden's Index, and Kappa values were calculated to identify the optimal predictive model. Calibration curves were also plotted to assess the predictive accuracy and clinical applicability of the selected model. All models were interpreted using the Shapley Additive Explanations (SHAP) method.

Statistical analyses were two-sided, with *p* < 0.05 considered statistically significant. All analyses were performed using R 4.4.2 statistical software and its integrated development environment, RStudio.

## Results

Table 1 presents the characteristics of the study population. A total of 14,492 participants were included in the analysis, among whom 1,212 (7.87%) were diagnosed with depression. The median age of the participants was 46 years, and 6,945 (49.85%) were male. No significant differences were observed between groups in terms of race, poverty-to-income ratio (PIR), or daily fiber intake. However, significant differences were found between the OSA and non-OSA groups for the following variables: age, sex, alcohol use, marital status, education level, smoking status, BMI, hypertension, diabetes, daily caffeine intake, daily fat intake, and depression.Compared to the non-OSA group, the OSA group had a higher proportion of individuals who were alcohol users, smokers, or diagnosed with hypertension, diabetes, or depression.

**Table 1:** Baseline Participant Characteristics

**Characteristic**	**N**^1^	**Overall** *N* = 176,571,036	**Obstructive Sleep Apnea(OSA)**		***P*-value**^2^
			**No** (***N* = **89,928,524)	**Yes** (*N* = 86,642,512)	
Age (years), Median (Q1, Q3)	14,492	46.00 (33.00, 59.00)	44.00 (30.00, 59.00)	48.00 (37.00, 60.00)	< 0.001
Sex, n(%)	14,492				< 0.001
Female		6,945.00 (49.85%)	3,865.00 (56.33%)	3,080.00 (43.12%)	
Male		7,547.00 (50.15%)	3,357.00 (43.67%)	4,190.00 (56.88%)	
Race, n(%)	14,492				0.087
Non-Hispanic White		6,596.00 (70.38%)	3,391.00 (71.31%)	3,205.00 (69.43%)	
Non-Hispanic Black		3,092.00 (10.21%)	1,556.00 (10.17%)	1,536.00 (10.26%)	
Mexican American		2,322.00 (8.00%)	1,078.00 (7.58%)	1,244.00 (8.45%)	
Other		1,246.00 (6.73%)	651.00 (6.74%)	595.00 (6.72%)	
Other Hispanic		1,236.00 (4.67%)	546.00 (4.21%)	690.00 (5.15%)	
Alcohol status, n(%)	14,492				< 0.001
No		12,072.00 (83.83%)	6,210.00 (86.96%)	5,862.00 (80.57%)	
Yes		2,420.00 (16.17%)	1,012.00 (13.04%)	1,408.00 (19.43%)	
Marital, n(%)	14,492				< 0.001
Married		7,585.00 (54.67%)	3,352.00 (49.71%)	4,233.00 (59.83%)	
Widowed		971.00 (5.16%)	593.00 (6.02%)	378.00 (4.26%)	
Divorced		1,700.00 (11.17%)	905.00 (11.80%)	795.00 (10.52%)	
Separated		500.00 (2.56%)	259.00 (2.53%)	241.00 (2.59%)	
Never married		2,414.00 (17.13%)	1,470.00 (21.07%)	944.00 (13.05%)	
Living with partner		1,322.00 (9.31%)	643.00 (8.86%)	679.00 (9.76%)	
Education, n(%)	14,492				0.003
Below high school		3,193.00 (13.22%)	1,493.00 (12.12%)	1,700.00 (14.35%)	
High school graduate		3,516.00 (24.90%)	1,717.00 (24.04%)	1,799.00 (25.79%)	
Above high school		7,783.00 (61.89%)	4,012.00 (63.84%)	3,771.00 (59.86%)	
Pir, n(%)	14,492				0.3
< = 1		2,633.00 (12.40%)	1,369.00 (12.99%)	1,264.00 (11.79%)	
> 3.0		5,689.00 (52.99%)	2,824.00 (52.52%)	2,865.00 (53.49%)	
1.1–3.0		6,170.00 (34.60%)	3,029.00 (34.49%)	3,141.00 (34.72%)	
Smoking status, n(%)	14,492				< 0.001
Smoker		7,361.00 (49.84%)	3,439.00 (45.85%)	3,922.00 (53.98%)	
No-smoker		7,131.00 (50.16%)	3,783.00 (54.15%)	3,348.00 (46.02%)	
BMI, n(%)	14,492				< 0.001
Underweight		221.00 (1.55%)	161.00 (2.21%)	60.00 (0.86%)	
Normal weight		3,788.00 (27.53%)	2,459.00 (36.13%)	1,329.00 (18.60%)	
Overweight		4,813.00 (32.67%)	2,416.00 (32.66%)	2,397.00 (32.68%)	
Obesity		5,670.00 (38.26%)	2,186.00 (29.00%)	3,484.00 (47.86%)	
Daily Fat intake, Median (Q1, Q3)	14,492	77.57 (56.30, 104.72)	74.31 (54.43, 100.14)	81.27 (58.47, 109.10)	< 0.001
Daily Fiber intake, Median (Q1, Q3)	14,492	15.00 (10.35, 20.95)	15.05 (10.15, 21.20)	15.00 (10.60, 20.80)	0.8
Daily Caffeine intake, Median (Q1, Q3)	14,492	137.00 (50.00, 252.50)	126.00 (42.50, 233.50)	146.50 (60.00, 273.00)	< 0.001
Hypertension, n(%)	14,492				< 0.001
Yes		5,110.00 (31.45%)	2,173.00 (26.26%)	2,937.00 (36.84%)	
No		9,382.00 (68.55%)	5,049.00 (73.74%)	4,333.00 (63.16%)	
Diabetes, n(%)	14,492				< 0.001
No		12,660.00 (90.84%)	6,481.00 (92.89%)	6,179.00 (88.71%)	
Yes		1,832.00 (9.16%)	741.00 (7.11%)	1,091.00 (11.29%)	
Depression, n(%)	14,492				0.001
No		13,280.00 (92.13%)	6,725.00 (93.17%)	6,555.00 (91.05%)	
Yes		1,212.00 (7.87%)	497.00 (6.83%)	715.00 (8.95%)	

^1^N not Missing (unweighted)

^2^Design-based KruskalWallis test; Pearson’s X^2: Rao & Scott adjustment

The results of the sample-weighted multivariable logistic regression analyses are presented in Table 2. A positive association between depression and OSA was observed across all models. The odds ratios (ORs) and 95% confidence intervals (CIs) were as follows:Model1:OR = 1.34 (95% CI:1.13–1.59),Model2:OR = 1.54(95%CI: 1.28–1.86) and Model 3: OR = 1.31 (95% CI: 1.08–1.60).

**Table 2:** Association between depression and OSA symptoms in weighted multivariable logistic regression

Variable	Model1 OR (95% CI)	*P*-value	Model2 OR (95% CI)	*P*-value	Model3 OR (95% CI)	*P*-value
Depression,no	1 (ref)	< 0.001	1 (ref)	< 0.001	1 (ref)	< 0.005
Depression,yes	1.34(1.13, 1.59)		1.54(1.28, 1.86)		1.31(1.08, 1.60	

*BMI* body mass index, *PIR* poverty income ratio, *OR* odds ratio, *CI* confidence interval, Ref reference

Model 1: crude model

Model 2: adjusted for age,sex,race,marital status,education,pir

Model 3: adjusted for age, sex, race, alcohol status, marital status, education, pir, smoking status, BMI, daily fat intake, daily fiber intake, daily caffeine intake, hypertension,diabetes

### Subgroup

**Fig. 2:**
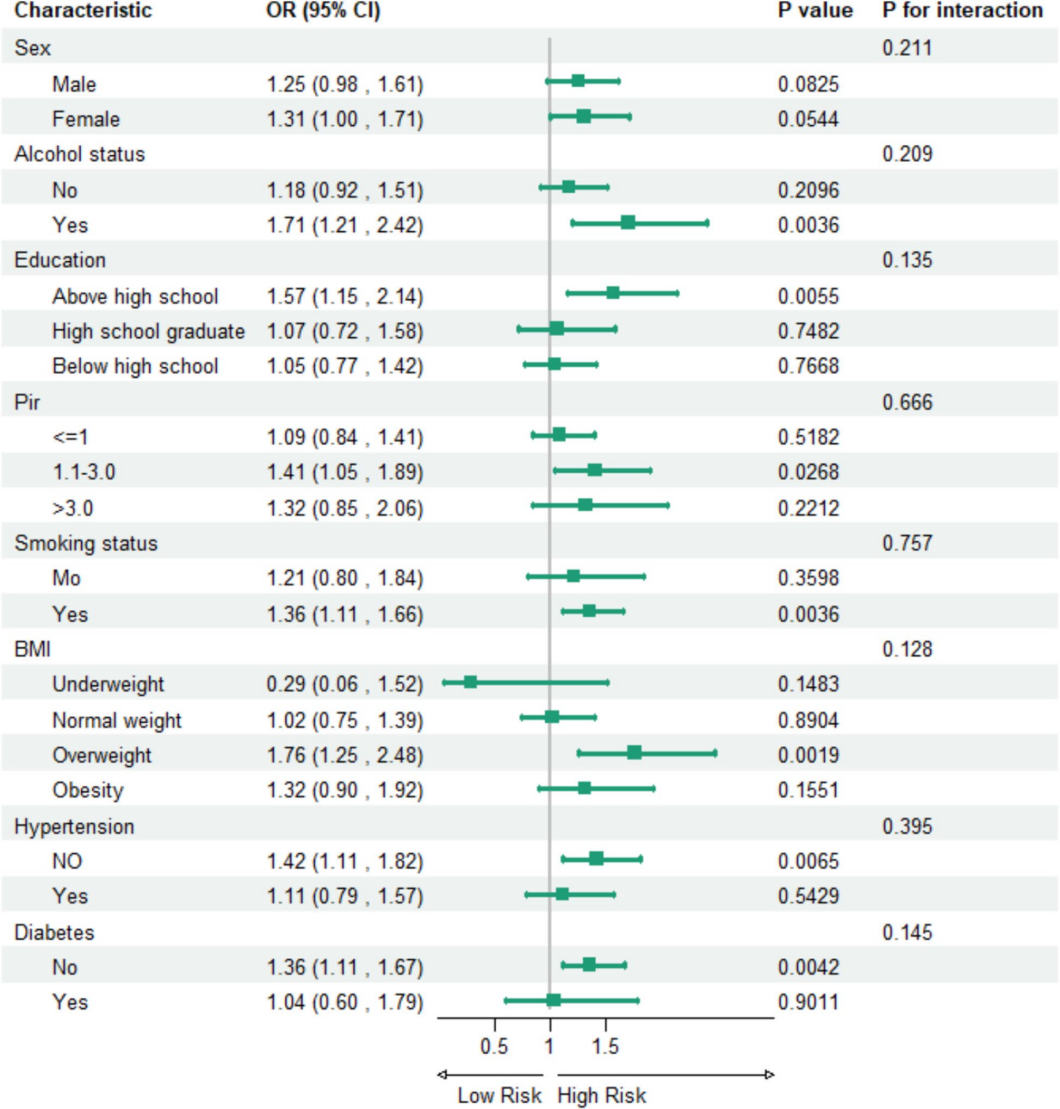
Multivariable odds ratios (ORs) for OSA based on depression status, stratified by sex, alcohol status, education, PIR, smoking status, BMI, hypertension, and diabetes, were adjusted for all factors within each stratification. OSA obstructive sleep apnea, BMI Body Mass Index, PIR poverty income ratio, OR odds ratio, CI Confidence interval

Figure 2 illustrates the relationship between depression and OSA through a comprehensive subgroup analysis. The analysis was conducted using fully adjusted multivariate logistic regression, accounting for potential confounding factors. The study population was stratified into subgroups based on sex, alcohol use, education level, PIR, smoking status, BMI, diabetes, and hypertension. No significant interaction effects were observed between the subgroups.

### Sensitivity analyses

Sensitivity analyses demonstrated the robustness of the primary findings.Comparison of excluded versus included participants revealed significant differences in demographic and clinical characteristics (all *p* < 0.001 except diabetes [*p* = 0.7]), suggesting potential selection bias (Table S1). Multiple imputation with inverse probability weighting strengthened the depression-OSA association (OR = 1.54, 95% CI 1.37–1.73) compared to the main model (OR = 1.31), indicating possible underestimation in the primary analysis(Table S2).After adjusting for insomnia and other covariates, depression remained significantly associated with OSA (OR = 1.25, 95%CI:1.03–1.53, *p* = 0.021). Stratified analyses revealed this association was stronger in participants without insomnia (OR = 1.65, 1.22–2.23) compared to those with insomnia (OR = 1.05, 0.79–1.40), though the formal interaction test was not significant (*p* = 0.11) (TableS3).

### A risk prediction model for OSA disease in depressed populations based on machine learning

**Fig. 3:**
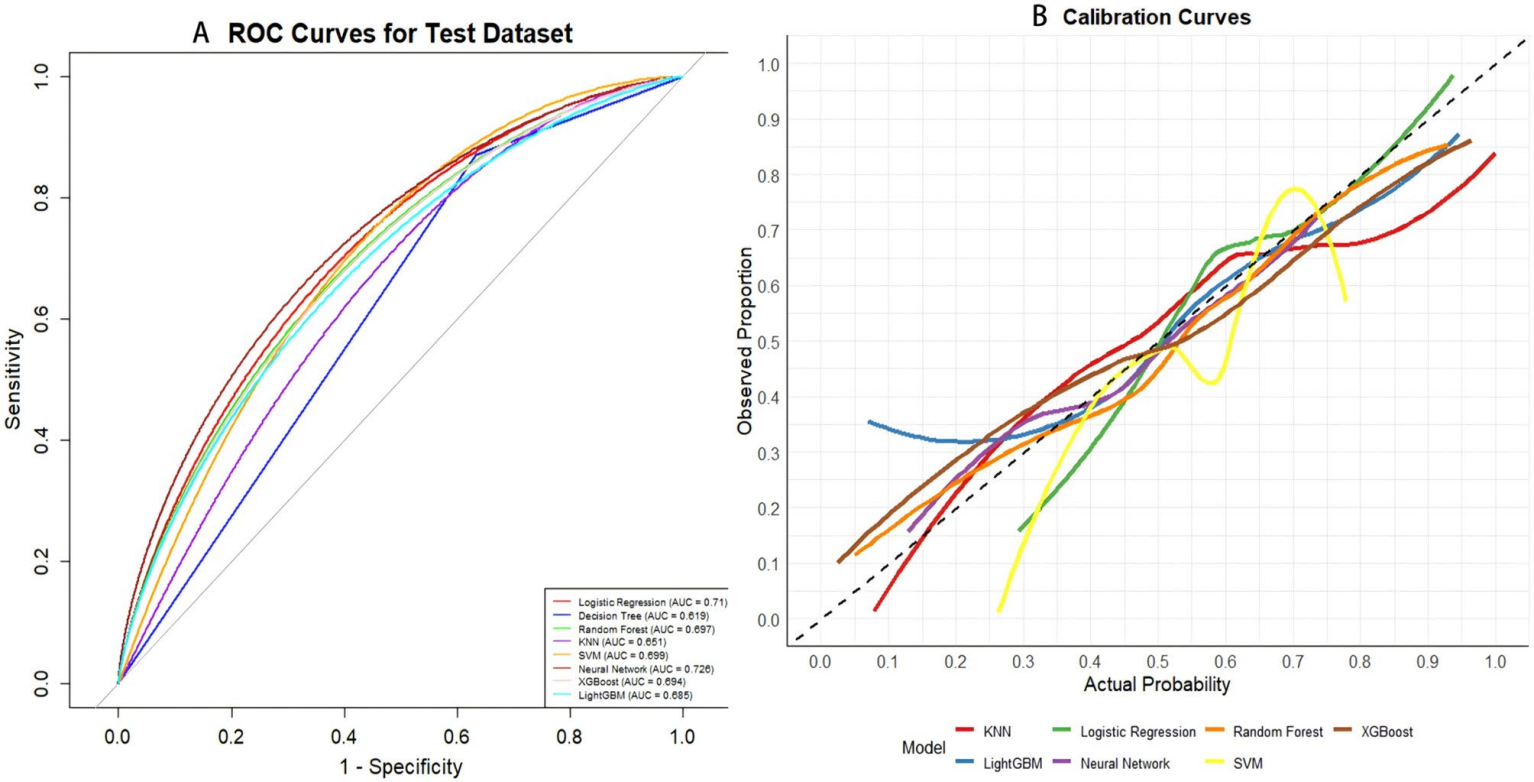
**A** ROC Curve for OSA Prediction: The receiver-operating characteristic (ROC) curve demonstrates the model's performance, achieving an Area Under the Curve (AUC) of 0.726. This indicates strong discriminative ability and stable generalization. **B** Calibration Curve for Validation Cohort: The calibration curve for the Neural Network (NNET) model closely aligns with the ideal line, outperforming other models in terms of predictive accuracy

Eight machine learning models were evaluated to predict OSA risk in a depressed population using test set data. The ROC curve results are shown in Fig. 3A. Among the models, the Neural Network (NNET) (AUC = 0.726) and Logistic Regression (LR) (AUC = 0.710) demonstrated the highest accuracy in identifying OSA risk, outperforming Decision Tree (DT) (0.619), Random Forest (RF) (0.697), K-Nearest Neighbors (KNN) (0.651), Support Vector Machine (SVM) (0.699), Extreme Gradient Boosting (XGBoost) (0.694), and Light Gradient Boosting Machine(LightGBM) (0.685). The confusion matrix parameters further confirmed the robustness of the models, as detailed in Supplementary Table S4.The calibration curves revealed that the NNET model outperformed the LR model, as illustrated in Fig. 3B. Based on these results and the applicability of the models, the Neural Network (NNET) was identified as the top-performing model, making it the most suitable for subsequent applications in OSA risk prediction and characterization.

### Interpretation of model features

**Fig. 4:**
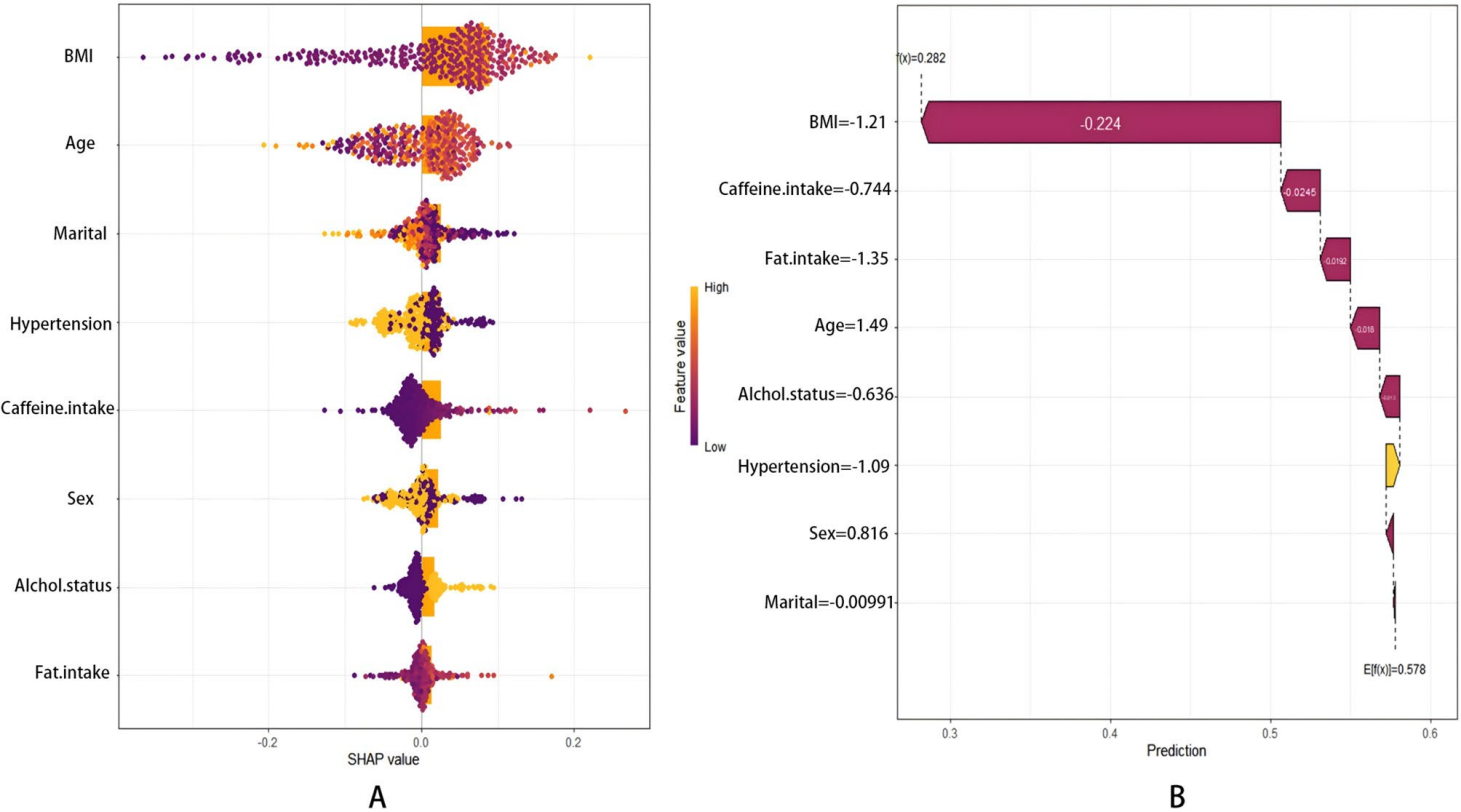
**A** SHAP Beehive Diagrams for the NNET model is presented with the horizontal axis (X-axis) representing the SHAP value for each sample, which indicates the degree of influence a feature has on the prediction.The vertical axis (Y-axis) enumerates the significant features of the model, ordered from to magnitude of a feature's contribution. Each dot represents the SHAP value of a sample, with yellow dots denoting high individual values and blue dots indicating low individual values. **B** demonstrates the model’s prediction result for the individual sample numbered 12

Figure 4A employs horizontal bar charts to visualize the contribution of each feature to the NEET model's predictions using SHAP values. The length of each bar represents the magnitude of a feature's contribution, while the Beehive Diagrams illustrates both the magnitude and direction of the impact of individual feature values on the predictions. The variables are ordered by their SHAP values, reflecting their relative importance in predicting OSA. The Beehive Diagrams further reveals how the internal characteristics of different variables influence OSA risk

In the SHAP summary plot (Fig. 4A), BMI, age, and marital status emerged as the top contributors to OSA prediction. BMI was identified as the most significant feature, exerting the strongest influence on the model's predictions. Age and marital status also played important roles, followed by hypertension and total caffeine intake, which demonstrated notable contributions to the model.

Figure 4B displays the model's prediction outcome for an individual sample (ID: 12), highlighting the magnitude and direction of each feature's contribution to the prediction.

## Discussion

This study examined the association between depression and the risk of OSA using data from the 2005–2008 and 2015–2018 NHANES cycles. The results indicate a positive relationship between depression and OSA risk after adjusting for potential confounding factors. Interaction tests revealed no statistically significant differences across subgroups stratified by sex, alcohol use, education level, PIR, smoking status,BMI, diabetes, or hypertension.

Within the depressed population, multiple machine learning models were developed and evaluated. Based on performance metrics and calibration curves, the NNET model was identified as the most suitable for predicting OSA risk. Furthermore, the SHAP method was employed to interpret the model, highlighting BMI as the most significant predictor, a finding consistent with clinical observations.

The relationship between depression and OSA remains a topic of debate. A bidirectional Mendelian randomization study identified a significant causal effect of depression on OSA [28].Additionally, research suggests that depressive symptoms are closely linked to both the presence and severity of OSA [29]. And BMI as an independent factor of OSA,it also have an influence on depression [30, 31].Obesity is commonly linked to neuroendocrine dysfunction, particularly in the HPA axis. Stress system activation may trigger mood disturbances, psychotic exacerbations, and cognitive impairment [32].The study supported that obesity and depression are potential risk factors on OSA risk [33]. However, findings from a large clinical cohort of individuals with suspected OSA revealed no association between OSA symptoms or severity and the risk of hospitalization for depression after adjusting for confounders[14]. Similarly, multivariate analyses in another study found no significant link between depression and OSA [15].

On the other hand, a prospective study demonstrated a clear association between OSA and the development of depressive disorders within one year, particularly among women [34]. These findings align with the results of our subgroup analysis. Data from the 2020 Korea National Health and Nutrition Examination Survey also support this relationship, showing that a higher risk of OSA is associated with an increased prevalence of depressive symptoms [9]. This contrasts with earlier analyses of NHANES data, which suggested that OSA is more prevalent among individuals with depression [35]. However, those analyses were limited to two cycles (2005–2008), whereas our study incorporates data from four cycles, enhancing the robustness of the findings.A key distinction of our study lies in its focus on the association between depression and OSA within a population adjusted for multiple confounding factors, a methodological approach that sets it apart from prior research.In our analysis, after adjusting for multiple confounding factors through multivariate logistic regression, depression was significantly and positively associated with OSA symptoms.

Numerous studies have underscored the connection between depression and OSA, yet the underlying mechanisms remain poorly understood. Emerging evidence suggests that disruptions in circadian clock gene expression among individuals with OSA may correlate with depressive symptoms [36]. Inflammatory factors are also hypothesized to play a mediating role in this relationship. For instance, elevated levels of inflammatory markers such as tumor necrosis factor-α (TNF-α) and interleukin-6 (IL-6) have been observed in OSA patients compared to healthy controls [37]. Similarly, increased concentrations of TNF-α and IL-6 have been reported in individuals with depressive disorders [19]. Another potential mechanism involves oxidative stress, which is triggered by intermittent hypoxia during sleep in OSA patients [20]. Notably, studies have found higher oxidative stress indices and reduced daily antioxidant capacity in individuals with depression compared to controls [38].Furthermore, research indicates that treating OSA can lead to improvements in cognitive symptoms [39]. Similarly, continuous positive airway pressure (CPAP) therapy has been shown to alleviate anxiety, depression, and sleep-related symptoms in OSA patients [40].

In summary, the application of machine learning (ML) algorithms to study the relationship between depression and OSA offers a promising avenue for identifying novel predictive factors and improving clinical decision-making. This study highlights the importance of model interpretability by leveraging explanatory tools to clarify the predictions of complex ML algorithms, thereby enhancing their clinical utility. Among the evaluated ML models, the Neural Network (NNET) achieved the highest Area Under the Curve (AUC). Although performance gains were modest,compared to logistic regression model, NNET was prioritized for its ability to model non-linear interactions that align with OSA's multifactorial pathophysiology. Using this model, BMI and age were identified as the most influential predictors of OSA, as reflected by their highest SHAP values. This finding simplifies the prediction process and facilitates its practical application in clinical settings.

Compared to traditional methods, the ML-based predictive model demonstrated superior performance metrics, underscoring its potential to refine diagnostic approaches for OSA in individuals with depression. Future research should focus on integrating diverse predictive indicators to further improve accuracy and support personalized treatment strategies, ultimately reducing disease progression and hospitalization rates.

This study has several limitations. First, the use of cross-sectional data precludes the establishment of causal relationships. Additionally, the diagnosis of both depression and OSA in the NHANES dataset is based on self-reported information, which may not always align with clinical diagnoses.Insomnia could partially mediate or obscure the depression-OSA link. This discrepancy could introduce misclassification bias and compromise the accuracy of the predictive models. Moreover, the selection of OSA-related variables from the NHANES data was guided by existing literature and researcher judgment, which may introduce subjectivity. Although our ML models employed regularization techniques, the modest sample size of depressed participants (*n* = 1212) may increase overfitting risk. External validation in larger cohorts is needed to confirm generalizability. SHAP results may not generalize to non-depressed populations.Finally,although the models exhibit performance metrics, their clinical applicability in real-world settings requires further validation by the broader scientific and medical community.

## Conclusion

In conclusion, our research suggests a potential association between depression and OSA, underscoring the importance of early detection and management of depression in individuals at risk of OSA.

By leveraging a comprehensive dataset, this study demonstrates the advantages of using machine learning (ML) models to predict OSA risk in individuals with depression. These models not only identify key risk factors but also provide valuable insights into the multifaceted nature of OSA. Furthermore, they highlight the critical role of considering various lifestyle, dietary, and health-related factors in understanding and addressing this complex condition.

## Supplementary Information

Supplementary Material 1.

## Data Availability

The datasets supporting the conclusions of this article are available in the National Health and Nutrition Examination Survey repository https://www.cdc.gov/nchs/nhanes/default.aspx.
