# Supplementary material for: Investigating the role of depression in obstructive sleep apnea and predicting risk factors for OSA in depressed patients: machine learning-assisted evidence from NHANES
Source: BMC Psychiatry. 2025 Oct 10;25:964. doi: 10.1186/s12888-025-07414-x (PMC12512373; doi:10.1186/s12888-025-07414-x)
Supplement: Supplementary file 1 — Supplementary Material 1. [file 12888_2025_7414_MOESM1_ESM.docx]

# TableS1 Baseline Characteristics

| **Characteristic** | **Excluded *by* Missing**  N = 5,924^1^ | **Final_Participants**  N = 14,492^1^ | **p-value**^2^ |
| --- | --- | --- | --- |
| **Age** | 44.9 (21.7) | 49.2 (17.5) | <0.001 |
| **Sex** |  |  | <0.001 |
| Female | 3,486 (59%) | 6,945 (48%) |  |
| Male | 2,438 (41%) | 7,547 (52%) |  |
| **Race** |  |  | <0.001 |
| Mexican American | 1,277 (22%) | 2,322 (16%) |  |
| Other Hispanic | 640 (11%) | 1,236 (8.5%) |  |
| Non-Hispanic White | 1,749 (30%) | 6,596 (46%) |  |
| Non-Hispanic Black | 1,446 (24%) | 3,092 (21%) |  |
| Other | 812 (14%) | 1,246 (8.6%) |  |
| **Alcohol status** | 328 (14%) | 2,420 (17%) | 0.006 |
| **Marital** |  |  | <0.001 |
| Married | 2,398 (46%) | 7,585 (52%) |  |
| Widowed | 540 (10%) | 971 (6.7%) |  |
| Divorced | 392 (7.6%) | 1,700 (12%) |  |
| Separated | 165 (3.2%) | 500 (3.5%) |  |
| Never married | 1,309 (25%) | 2,414 (17%) |  |
| Living with partner | 363 (7.0%) | 1,322 (9.1%) |  |
| **Education** |  |  | <0.001 |
| Below high school | 1,536 (33%) | 3,193 (22%) |  |
| High school graduate | 1,036 (22%) | 3,516 (24%) |  |
| Above high school | 2,078 (45%) | 7,783 (54%) |  |
| **Pir** |  |  | <0.001 |
| <=1 | 1,254 (31%) | 2,633 (18%) |  |
| >3.0 | 1,036 (25%) | 5,689 (39%) |  |
| 1.1-3.0 | 1,817 (44%) | 6,170 (43%) |  |
| **Smoking status** |  |  | <0.001 |
| Smoker | 1,371 (27%) | 7,361 (51%) |  |
| No-smoker | 3,769 (73%) | 7,131 (49%) |  |
| **BMI** |  |  | <0.001 |
| Underweight | 139 (2.4%) | 221 (1.5%) |  |
| Normal weight | 1,832 (32%) | 3,788 (26%) |  |
| Overweight | 1,777 (31%) | 4,813 (33%) |  |
| Obesity | 1,956 (34%) | 5,670 (39%) |  |
| **Daily fiber intake** | 15.8 (8.9) | 16.4 (9.1) | <0.001 |
| **Daily fat intake** | 71.9 (38.6) | 81.4 (41.0) | <0.001 |
| **Daily Caffeine intake** | 88.7 (133.5) | 156.4 (192.4) | <0.001 |
| **Hypertension)** |  |  | <0.001 |
| Yes | 1,791 (30%) | 5,110 (35%) |  |
| No | 4,104 (70%) | 9,382 (65%) |  |
| **Diabetes** | 757 (13%) | 1,832 (13%) | 0.7 |
| ^1^Mean (SD); n (%) | | | |
| ^2^Wilcoxon rank sum test; Pearson's Chi-squared test | | | |

# Table S2 Association Between Depression and Obstructive Sleep Apnea After Multiple Imputation and Inverse Probability Weighting

| **Characteristic** | **OR^1^** | **95% CI^1^** | **p-value** |
| --- | --- | --- | --- |
| **Depression** |  |  | <0.001 |
| **No** | — | — |  |
| **Yes** | 1.54 | (1.37–1.73) |  |
| Analysis based on 5 imputed datasets with IPW adjustment for age, sex, race, marital status, education, smoking, PIR, BMI, dietary factors, hypertension, and alcohol use. | | | |

# Table S3 Multivariable Analysis of Depression and Insomnia on OSA Risk

| **Characteristic** | **OR (95% CI)** | **p-value** |
| --- | --- | --- |
| **Depression Status** |  | 0.021 |
| **No (Ref)** | 1.00 | — |
| **Yes** | 1.25 (1.03–1.53) |  |
| **Stratified Analysis** |  |  |
| **No Insomnia** | 1.65 (1.22–2.23) | 0.003 |
| **Insomnia** | 1.05 (0.79–1.40) | 0.722 |
| **Interaction** | 0.69 (0.44–1.09) | 0.11 |
| OR: Odds Ratio; CI: Confidence Interval  **Main Model**: Adjusted for insomnia and all covariates (age, sex, race, etc.)  **Stratified Models**: Adjusted for the same covariates excluding insomnia  **Interaction Test**: p-value for multiplicative interaction term (depression × insomnia) in the fully adjusted model | | |

# Table S4: Performance metrics of different machine learning approaches

| **Model** | **Accuracy** | **Sensitivity** | **Specificity** | **PPV** | **NPV** | **AUC** | **Youden's Index** | **Kappa** |
| --- | --- | --- | --- | --- | --- | --- | --- | --- |
| **LR** | 0.6722 | 0.8143 | 0.4771 | 0.6813 | 0.6518 | 0.71 | 0.2914 | 0.3024 |
| **DT** | 0.6584 | 0.8714 | 0.3660 | 0.6536 | 0.6747 | 0.619 | 0.2374 | 0.2532 |
| **RF** | 0.6832 | 0.8429 | 0.4641 | 0.6834 | 0.6827 | 0.697 | 0.307 | 0.3209 |
| **KNN** | 0.6501 | 0.8095 | 0.4314 | 0.6615 | 0.6226 | 0.651 | 0.2409 | 0.2514 |
| **SVM** | 0.6446 | 0.8524 | 0.3595 | 0.6462 | 0.6395 | 0.699 | 0.2119 | 0.2252 |
| **NN** | 0.6804 | 0.7333 | 0.6078 | 0.7196 | 0.6242 | 0.726 | 0.3411 | 0.3424 |
| **XG Boost** | 0.6501 | 0.7905 | 0.4575 | 0.6667 | 0.6140 | 0.694 | 0.248 | 0.2569 |
| **LG** | 0.6639 | 0.8000 | 0.4771 | 0.6774 | 0.6348 | 0.685 | 0.2771 | 0.2868 |
| **Abbreviation**: AUC, Area Under the Curve; LR, Logistic Regression; DT,Decision Tree;RF, Random Forest; KNN,K-Nearest Neighbors;SVM, Support Vector Machine;XG Boost, eXtreme Gradient Boost; Light-GBM, Light Gradient Boosted Machine;NPV:Negative Predictive Value,PPV:Positive Predictive Val | | | | | | | | |
